# Overuse and underuse of cardiovascular diagnostic and therapeutic procedures for community-dwelling adults: a protocol for a systematic review

**DOI:** 10.12688/hrbopenres.13330.1

**Published:** 2021-09-02

**Authors:** Dominic Quinn, David Byrne, Tom Fahey, Rose Anne Kenny, Christine McGarrigle, Emma Wallace, Fiona Boland

**Affiliations:** 1 The Irish Longitudinal Study on Ageing (TILDA), Trinity College Dublin, the University of Dublin, Dublin, Ireland; 2 HRB Centre for Primary Care Research, Department of General Practice, Royal College of Surgeons in Ireland, Dublin, Ireland; 3 Data Science Centre, Royal College of Surgeons in Ireland, Dublin, Ireland

**Keywords:** health care utilisation, potentially inappropriate, overuse, underuse, cardiovascular procedures.

## Abstract

**Background: **Potentially inappropriate care can result from overuse or underuse of treatments, tests, or procedures. Overuse is defined as the use of health services with no clear benefit to the recipient or where harms outweigh benefits and/or costs of care. Underuse is defined as failure to deliver an effective and cost-effective healthcare intervention. Cardiovascular procedures such as coronary artery bypass grafting, carotid endarterectomy, coronary angiography, and coronary angioplasty (with/without stenting) are potentially both underused and overused. This systematic review aims to identify rates of potential overuse and underuse of these cardiovascular procedures and explore any associated patient or healthcare system factors.

**Methods:** A systematic review and meta-analysis will be conducted in accordance with the PRISMA guidelines. A systematic search of MEDLINE (via Ovid), Embase, Cumulative Index to Nursing and Allied Health Literature and the Cochrane library will be conducted using a predefined search strategy.  Eligible studies for inclusion will examine rates of overuse and underuse of cardiovascular procedures, measured against national/international guidelines, for adults aged ≥18 years. Primary observational studies including cross-sectional and cohort studies will be included. Titles, abstracts, and full texts will be screened for inclusion by two reviewers. Data will be extracted using a standardised form. Risk of bias for all included studies will be assessed using a modified version of the Hoy risk of bias tool. Where adequate data exists, and if statistically appropriate, meta-analyses will be conducted. If statistical pooling of the data is not possible, the findings will be narratively summarised focusing on the review’s objectives.

**Conclusion**: This systematic review will examine overuse and underuse of cardiovascular procedures for adults.  The results will help inform policy makers, researchers, patients, and clinicians in the appropriate use of these procedures, in line with international guidelines.

**Registration: **This protocol has been submitted for registration on PROSPERO (CRD42021239041).

## Introduction

Variation in healthcare can occur for various complex and inter-related reasons ^1^. Examples of variation include geographical, gender or age differences, patient or clinician preference in relation to conditions where more than one treatment option exists (preference-sensitive care), or availability and access to particular treatments or services (supply-sensitive care) ^1, 2^. Certain variation in healthcare can be expected and can sometimes be appropriate in reflecting the differences in health care needs across different populations and new or emerging evidence ^3^. However, other variation may be unwarranted, resulting from potentially inappropriate care. Clinical guidelines are evidence-based recommendations on the appropriate care and treatment of people (e.g. National Institute for Health and Care Excellence (NICE) guidelines). Despite some recognised limitations, including varying quality of guidelines, guidelines are often used as markers of healthcare appropriateness ^4^.

Potentially inappropriate care can result from overuse or underuse of treatments, tests, or procedures ^5^. Overuse is defined as the use of health services without clear benefit or where harms outweigh benefits and/or costs, and underuse is defined as the failure to deliver an effective and cost-effective medical intervention ^6, 7^. There are several potential drivers of overuse and examples include greater access to treatments or services, a desire to fulfil patients’ expectations or fear of litigation ^4, 8^. Furthermore, increasing workload and insufficient time during consultations or difficulty keeping up-to-date with rapidly changing evidence may result in overuse or underuse of healthcare. Overuse and underuse can have significant consequences for patients. Overuse can subject patients to direct harms or result in incidental findings and overdiagnosis, and can also result in resource implications, increasing the use of healthcare resources, and increasing healthcare expenditure. On the contrary, underuse can result in delays in diagnosis and delivery of effective treatments ^4^.

Cardiovascular disease is among the leading causes of mortality rates in the world ^4^. Rates of potentially inappropriate use of cardiovascular procedures such as coronary artery bypass grafting (CABG), carotid endarterectomy (CEA), coronary angiography (CA) and coronary angioplasty (with/without stenting) vary in the literature. CEA is an example of a surgical procedure where the risks versus benefits ratio is unclear and has the greatest variation of frequency, up to four-fold both within and outside of the United States of America (USA) ^9^. In 2004, a Canadian study assessed CEA procedures (n=3167) using the RAND-UCLA appropriateness method and found that approximately one in 10 were potentially inappropriate ^10^. In an American study, conducted in New York, the rate of potentially inappropriate CEA was 8.4% across Medicare Health Maintenance Organisations (HMO) and 8.6% in Medicare Fee-for-service (FFS) plans ^11^. In a national US study, the rate of potentially inappropriate CA was 13% across both Medicare HMOs and Medicare FFS plans ^12^. A small number of systematic reviews, conducted mainly in the US, examined the rate of potential overuse of these procedures that ranged from 1.4%-21.8% (CA 8%-21.8%, CEA 8.6%-10.6%, and coronary revascularisation 1.4%-14%) in studies (n=48) published from 2000 onwards ^6, 13– 16^.

While the aforementioned studies tend to focus on potentially inappropriate overuse, procedures can be both inappropriately overused and underused. A retrospective study carried out by Ko and colleagues ^17^ revealed underuse of coronary revascularisation procedures, with 31% of patients deemed eligible to undergo coronary revascularisation procedure not receiving the procedure. Expert panels have identified underuse of cardiovascular procedures ranging from 21% to 42% in patients with coronary artery disease where intervention is deemed appropriate ^9, 18– 22^. Among 9,458 Medicare patients with acute myocardial infarction, 42% did not undergo CA according to guidelines recommendations. An American study conducted using the RAND-UCLA appropriateness method ^23^ across four public hospitals and two private hospitals in Los Angeles found that 26% of patients who underwent CA (n=107), and where percutaneous transluminal coronary angioplasty (PTCA) was deemed necessary, did not receive the procedure ^20^. A prospective study using expert consensus methodology conducted in three London hospitals found that in a total of 908 participants where PTCA was deemed appropriate, only 36% (n=327) of patients received this treatment while the remaining patients received either CABG or received medical treatment only (30% and 34%, respectively) ^18^.

This systematic review will address the following research objectives;

1. Identify rates of potential overuse and potential underuse of the following cardiovascular procedures; CABG, CEA, CA, and coronary angioplasty (with/without stenting), for community-dwelling adults (aged ≥18 years) benchmarked against national or international guidelines.
2. To identify and explore potential risk factors (e.g. patient characteristics, system characteristics) associated with over or underuse of these cardiovascular procedures.

## Protocol

The review will be conducted according to the Preferred Reporting Items for Systematic Reviews and Meta-Analysis (PRISMA) standardized reporting guidelines ^24^, and this protocol has been prepared in adherence to the PRISMA-Protocols (PRISMA-P) statement ^25^. This protocol has been submitted for registration on PROSPERO (CRD42021239041).

### Eligibility criteria

Eligibility criteria are outlined in Table 1. There will be no restrictions in terms of language but only articles published from 2000 onwards will be included to reflect current practice. If non-English articles are identified in the search, in the first instance Google translate will be used to assess potential eligibility. To accurately assess studies and findings, we will contact the authors and explore if the article has been previously translated to English. We will assess the number and language of identified studies and where possible invite collaborators to assist in assessing the studies and extracting data.

**Table 1.** Inclusion and exclusion criteria.

PROS strategy	Inclusion criteria	Exclusion criteria
P – Population	Adults aged ≥18 years.	Studies consisting of children and adolescents aged <18 years. We will exclude studies if they meet the following criteria: >20% of participants were children (>20% under 18 years old).
R – Risk factors	Any associated patient or healthcare system factor explored.	
O - Outcomes	Rates of potential inappropriateness (overuse or underuse) of cardiovascular therapeutic procedures namely, coronary angiography, coronary angioplasty (with/without stenting), coronary artery bypass grafting and carotid endarterectomy, measured against national or international guidelines.	Studies that do not explain how appropriateness was measured or use local/regional guidelines, such as guidelines specific to a hospital or region, rather than international or national guidelines.
S – Study design	Cross-sectional studies and cohort studies	Randomised controlled trials, non- randomised controlled trials, case studies, case series and qualitative studies, protocol studies, editorials, letters.

In terms of the outcome, a potential limitation is relying on authors to identify and include details on national/international recommended guidelines. Specifically, in terms of national guidelines, it may not be feasible to assess the quality and evidence behind each identified recommended guideline. In line with other systematic reviews on potentially inappropriate health care ^26^, we will document all guidelines provided in the studies.

### Information sources

Cohort and cross-sectional studies will be retrieved using the following databases: MEDLINE (via Ovid), Embase, Cumulative Index to Nursing and Allied Health Literature, and the Cochrane library. OpenGrey and Google Scholar will be searched for grey literature.

### Search strategy

Search strings for the systematic review have been developed with dedicated Academic Librarian support. A combination of key words and MeSH terms will be included and can be summarised as `Ambulatory care AND adherence AND guidelines AND cardiovascular therapeutic procedures AND inappropriate’. A sample search strategy for Medline is presented in extended data and this will be adapted for all other databases ^27^.

## Study design

### Data management

References will be managed with Endnote X9 reference manager. All search results will be imported into EndNote and duplicates will be screened and removed.

### Selection process

Two reviewers (DQ and DB) will independently review all potentially eligible titles and/or abstracts of identified records, followed by full texts of studies considered eligible for inclusion. Discrepancies will be resolved by consensus or an independent third reviewer (FB or EW). A PRISMA flow diagram will be completed for the selection process recording reasons for exclusion of excluded articles for all potentially relevant articles.

### Data collection and extraction

Two reviewers (DQ and DB) will use a standardised, pre-piloted form (see extended data ^27^) to extract data on the following: study ID, authors, year of publication, dates of study, inclusion and exclusion criteria, country of study, disease and/or symptom being studied, cardiovascular procedure, number of patients and other patient demographics, name and type (national/international) of guidelines measuring appropriateness, guideline reference, name of guideline-issuing authority (and country), year of guideline publication, guideline recommendations, method of primary study data collection, number of patients that potentially inappropriately received the cardiovascular procedure (overuse), and/or number of patients that potentially inappropriately did not receive the cardiovascular procedure (underuse). Any potential risk or protective factors explored in association with rates of potential inappropriateness (overuse or underuse) of the cardiovascular therapeutic procedures and effect sizes will also be extracted. Any disagreements in the data extraction will be resolved by consensus or an independent third person (FB or EW). If a study presents ambiguous, incomplete or missing data, we will contact the study authors to acquire the appropriate data. The extent of missing data will be documented in the extraction form.

### Quality assessment

Studies will be included if they meet all inclusion criteria irrespective of quality. This risk of bias of all individual studies will be assessed using a modified version of the Hoy risk of bias tool ^28^. This tool has been validated to assess internal and external validity of prevalence studies ^28^. The wording of the tool will be altered to reflect prevalence of potentially inappropriate procedures but the domains from the tool will be retained. The tool assesses external validity of the study (selection and nonresponse bias) and internal validity (measurement bias and analysis bias). Each study will be graded low, moderate, or high risk of bias. The Grading of Recommendations, Assessment, Development and Evaluations assessment tool will be used to rate the quality of scientific evidence and present the evidence summary for the outcomes ^29^.

### Data synthesis and analysis

Cohort and cross-sectional studies will be reported separately. Descriptive statistics will be used initially. A potential challenge of this systematic review is the likely heterogeneity between studies in how potentially inappropriate care is defined and measured, which may limit our ability to statistically combine and compare studies. Where adequate data exists, and if statistically appropriate, data will be pooled together and analysed using a random-effects model to obtain summary effect estimate, 95% confidence interval and *p*-value. Heterogeneity between comparable studies will be explored through visual inspection of the forest plots, using the *χ* ^2^ test and *I ^2^* statistic. We will interpret an *I ^2^* value of 0% as an indication of no observed inconsistency/heterogeneity, 30%–60% as may represent moderate heterogeneity, 50%–90% as may represent substantial heterogeneity and 75% to 100% as considerable heterogeneity ^30^. Publication bias will be assessed using a funnel plot if ten or more studies are identified. Furthermore, sensitivity analysis will be conducted, excluding high risk of bias studies, to explore the impact on summary effect sizes. Review Manager (RevMan) 5.3 ^31^ or Stata version 16 ^32^ will be used. If statistical pooling of the data is not possible, the findings will be narratively summarised focusing on the review’s objectives.

### Dissemination of information

The review will be published in a peer-reviewed journal, reported in line with the PRISMA guidelines ^24^. The review will also be presented at relevant conferences.

### Study status

At time of publication the study is ongoing, and title and abstract screening is underway. It is anticipated that data collection and analysis should be complete by October 2021.

## Discussion

This review will systematically examine the available evidence on overuse and underuse of cardiovascular procedures, specifically CABG, CEA, CA and coronary angioplasty, and associated risk factors. To the best of our knowledge this will be the first systematic assessment focused on cardiovascular procedures. This systematic review will contribute to the evidence base for potentially inappropriate health care utilisation and could help inform policy makers, researchers, patients and clinicians in identifying specific potentially inappropriate cardiovascular procedures.

## Data availability

### Underlying data

No underlying data are associated with this article.

### Extended Data

Open Science Framework: Overuse and underuse of cardiovascular diagnostic and therapeutic procedures for community-dwelling adults: a protocol for a systematic review. https://doi.org/10.17605/OSF.IO/D8EWM ^28^

This project contains the following extended data:

- Medline Search Strategy.pdf
  A mix of key words and mesh terms that will be used to search Medline and that will be transferred to other databases.
- Data Extraction Template.pdf
  A draft data extraction template outlining headings under which study characteristics will be extracted.

### Reporting guidelines

PRISMA-P checklist for ‘Overuse and underuse of cardiovascular diagnostic and therapeutic procedures for community-dwelling adults: a protocol for a systematic review’. https://doi.org/10.17605/OSF.IO/D8EWM ^28^

Data are available under the terms of the Creative Commons Zero “No rights reserved” data waiver (CC0 1.0 Public domain dedication).
