# Supplementary material for: Overuse and underuse of cardiovascular diagnostic and therapeutic procedures for community-dwelling adults: a protocol for a systematic review
Source: HRB Open Res. 2021 Sep 2;4:99. [Version 1] doi: 10.12688/hrbopenres.13330.1 (PMC8976182; doi:10.12688/hrbopenres.13330.1)
Supplement: Peer review history

# Peer review: Reviewer response for version 1

**DOI:** 10.21956/hrbopenres.14514.r31553

**Published:** 2022-04-01

**Authors:** Teresa Pérez Pérez, Nieves Plana

**Affiliations:** 1 Departamento de Estadística y Ciencia de los Datos, Universidad Complutense de Madrid, Madrid, Spain; 2 Unidad de Evaluación de Tecnología Sanitaria, Hospital Ramón y Cajal, Madrid, Spain

Authors have designed a well-written protocol to answer a relevant question about the extent of the overuse and underuse of cardiovascular procedures for community-dwelling adults. There is growing awareness against the overuse and underuse of health care services. We have some minor comments:

**Search strategy**:

 What is the purpose to perform a search in the Cochrane Library for identify primary studies if you don´t consider to include randomized clinical trials in the review?

**Quality assessment:**

 The GRADE approach was developed to assess the certainty of the evidence in effects of interventions by outcome across studies. Although, there is no formal guidance for GRADE in systematic reviews of prevalence studies, there is some guidance into the use of GRADE for prognosis studies, which may be useful for your review (Iorio A *, et al. *2015 ^1^) *.* Have you consider performing that extended GRADE approach?

**Statistical analysis:**

 Random‐effects meta‐analysis model is frequently used to combine single proportions. For using the model, proportions are generally transformed using either the log, logit, arcsine, or the Freeman‐Tukey double arcsine transformations. Consider adding a line to briefly explain the method considered.

**Publication bias:**

 There is no evidence that proportional data adequately adjusts for Begg’s and Egger’s test (via funnel plot asymmetry) to evaluate publication bias. Some authors do not recommend its use and advise that publication bias be assessed qualitatively. Consider to include a comment about this limitation.

Is the study design appropriate for the research question?

Yes

Is the rationale for, and objectives of, the study clearly described?

Yes

Are sufficient details of the methods provided to allow replication by others?

Yes

Are the datasets clearly presented in a useable and accessible format?

Yes

Reviewer Expertise:

Medical statistics, epidemiology, evidence based medicine, health technology

We confirm that we have read this submission and believe that we have an appropriate level of expertise to confirm that it is of an acceptable scientific standard.

## References

[1] : Use of GRADE for assessment of evidence about prognosis: rating confidence in estimates of event rates in broad categories of patients. *BMJ* .2015;350: 10.1136/bmj.h870 h870 10.1136/bmj.h870 25775931

# Peer review: Reviewer response for version 1

**DOI:** 10.21956/hrbopenres.14514.r31398

**Published:** 2022-02-11

**Authors:** Stefano Ballestri

**Affiliations:** 1 Internal Medicine Unit, Pavullo Hospital, Modena, Italy

The Authors describe a protocol for a systematic review on overuse and underuse of cardiovascular diagnostic and therapeutic procedures for community-dwelling adults. This is a very hot topic and systematic reviews in the field are eagerly awaited. The study protocol is clear, well-written, and scientifically sound. 

 I have only one comment about the title to avoid misleading. I would remove "diagnostic" from the title, given that the review will systematically examine cardiovascular procedures, specifically CABG, CEA, CA, and coronary angioplasty. The only exception is coronary angiography which could be only diagnostic, but the review is mainly focused on therapeutic procedures.

Is the study design appropriate for the research question?

Yes

Is the rationale for, and objectives of, the study clearly described?

Yes

Are sufficient details of the methods provided to allow replication by others?

Yes

Are the datasets clearly presented in a useable and accessible format?

Yes

Reviewer Expertise:

Cardiovascular disease and liver, Metabolic Syndrome, Direct Oral Anticoagulants, Ultrasound

I confirm that I have read this submission and believe that I have an appropriate level of expertise to confirm that it is of an acceptable scientific standard.
